# Nutritional deficiencies and abortions in sheep and goats: An in-depth study from East Azerbaijan Province, Northwest Iran

**DOI:** 10.1371/journal.pone.0327768

**Published:** 2025-08-05

**Authors:** Hassan Sadri, Monireh Khordadmehr, Hamid Akbari, Jafar Shirazi, Yaser Jafari-Khataylou, Saba Eskandari, Bahareh Sadat Mirarabshahi, Ali Abdolmaleki

**Affiliations:** 1 Department of Clinical Sciences, Faculty of Veterinary Medicine, University of Tabriz, Tabriz, Iran; 2 Department of Pathobiology, Faculty of Veterinary Medicine, University of Tabriz, Tabriz, Iran; 3 Veterinary Organization, Tabriz, East Azerbaijan Province, Iran; UPR: University of the Poonch Rawalakot, PAKISTAN

## Abstract

Vitamin and mineral levels in sheep and goat herds experiencing abortions in East Azerbaijan, northwest Iran, were studied. Between November 2023 and February 2024, 373 blood samples and 62 samples from aborted fetuses in various cities were collected. To find out whether a lack of selenium and copper in mothers led to heart and brain problems in their fetuses. Sheep and goats were mainly raised in a semi-intensive system, grazing from spring to mid-autumn and keeping indoors during winter. Sheep and goat flocks were categorized by size: small (1–100 sheep), medium (101–300 sheep), and large (over 300 sheep). The data show significant deficiencies in essential vitamins and minerals, affecting animal health and reproduction. A notable lack of vitamin A was observed in Bostan Abad. Widespread vitamin D deficiency was noted, especially severe in Jolfa, suggesting diet inadequacies despite enough sunlight. A slight deficiency of vitamin E was found, alleviated through farmers’ supplements helped some. Calcium and phosphorus deficiencies, particularly calcium, were also major concerns. Copper and zinc shortages were common across different cities. Aborted fetuses from copper-deficient mothers showed brain tissue damage, like Wallerian degeneration and neuronal necrosis. Severe iodine deficiency was observed in Marand and Khoda Afarin, risking thyroid and reproductive health and function. More than 87% of samples revealed significant selenium deficiency, indicating a need for supplementation. Pathological studies showed heart tissue damage in aborted fetuses from selenium-deficient mothers, including fragmentation, calcification, and necrosis. These results highlight the need for proper nutritional interventions and regular monitoring of vitamin and mineral levels to fix deficiencies. Proper nutrition in mothers is crucial for lowering abortion risks and fetal developmental issues. Our data highly recommend dietary changes and balanced vitamin and mineral supplements in the studied province, considering local factors such as soil quality, pasture, and crop residues.

## Introduction

Sheep have significantly influenced human history, as one of the first animals to be domesticated around 9,000 years ago [1]. Today, sheep farming remains an economically important activity. For small-scale farmers, sheep are not only a source of income but also provide food, materials, and cultural benefits [2,3], particularly for nomadic and rural communities in regions such as Iran. However, issues such as abortion in sheep pose serious challenges, affecting food production, animal welfare, and the agricultural economy. Abortions lead to economic losses and public health risks due to both direct and indirect costs, including longer lambing intervals, higher feed costs, reduced income from lamb sales, fewer replacements, and higher veterinary costs [4,5]. The traditional nature of sheep farming in Iran, characterized by nomadic and semi-nomadic herding, seasonal migrations, decentralized breeding practices, and limited access to veterinary services poses significant challenges to implementing effective abortion prevention programs. Abortion in sheep is a significant concern for producers and veterinary departments, as it can drastically reduce flock productivity and result in substantial economic losses [4,6,7].

The causes of abortion can be broadly categorized into infectious and non-infectious factors [7], with nutritional deficiencies being a key non-infectious contributo. These deficiencies may encompass a lack of energy, macro- and micronutrients, and vitamins [4,8]. Feed availability and quality directly influence abortion rates, as adequate nutrient intake enhances reproductive success, especially during pregnancy [9]. Increased energy and nutrient supply improve hormonal functions necessary for ovulation [10]. Vitamins A, D, and E, along with minerals such as calcium, phosphorus, copper, selenium, iodine, and zinc, are critical for successful reproduction [11–13]. Deficiencies in these essential nutrients during pregnancy can lead to low birth weights and stillbirths, highlighting the importance of proper nutrition [4,14,15]. A thorough understanding of nutrition and reproductive health is vital for developing management practices aimed at reducing abortion rates. Studies addressing nutrient deficiencies and their impact on abortion in sheep in different countries suggest that such data can be instrumental in preventing or reducing the incidence of abortions [16,17]. However, an in-depth investigation specifically examining vitamin, macroelement and trace element deficiencies related to abortions in sheep and goats in Iran, particularly in East Azerbaijan Province, has yet to be conducted. It was hypothesized that deficiencies in vitamins, macro, and trace elements are significantly associated with increased abortion rates in sheep and goats in East Azerbaijan Province, Iran. East Azerbaijan Province is one of the leading regions in the country for sheep production. Gathering this data will be essential for preventing or reducing abortion rates in sheep and goat flocks, ultimately enhancing the productivity and sustainability of sheep farming.

## Materials and methods

### Study area, data collection, and sampling

This study was conducted in the cities of Tabriz, Marand, Charuymaq, Khoda Afarin, Jolfa, Heris, Bostan Abad, Mianeh, and Hashtrud, located in the East Azerbaijan province of northwest Iran. All relevant international, national, and institutional guidelines for the care and use of animals were followed, including the protocol approved by the Animal Research Ethics Committee of the University of Tabriz, Iran (ID: IR.TABRIZU.REC.1403.049). The findings focus on serum concentrations of vitamins, as well as macroelements and trace elements, as part of a larger investigation into the infectious and non-infectious causes of abortion in small ruminants in this region. A total of 373 blood samples were collected from local breeds of sheep and goats (Ghezel, Moghani, and Afshari) in these areas between November 2023 and February 2024. This was in response to reports of aborted (dead) fetuses from livestock owners; therefore, there was neither sacrifice nor any anesthesia or analgesia administered. Additionally, no protected species were included in the sampling. In the studied region, semi-intensive production systems are the most prevalent, combining agricultural production with sheep and goat farming. From spring to mid-autumn, the animals graze in pastures, while during the winter months, they are housed indoors and fed a diet mainly composed of forage, crop residues, and sometimes cereal grains, primarily wheat or barley. We analyzed 43 sheep flocks, documenting the history of the sampled herds, which included details such as herd size and abortion rate (S1 Table). The sheep flocks were all privately owned and unprotected. They were categorized by size: 7 small flocks (1–100 sheep), 19 medium flocks (101–300 sheep), and 17 large flocks (over 300 sheep).

Five mL of blood samples without anticoagulant were collected from both aborted and pregnant animals for analysis. The blood was centrifuged at 6000 rpm for 10 minutes to separate the serum. The serum was then transferred to sterile 1.5-mL microcentrifuge tubes and stored at −70 °C for further analysis.

### Measurement of vitamins and minerals

Measurements of vitamins A and E were performed using high-performance liquid chromatography (HPLC). The blood serum sample was thawed on ice, and 100 μL was transferred to a glass tube. Next, 2 mL of n-hexane was added, and the mixture was hand-mixed by inversion for 1 minute. It was then centrifuged for 5 minutes at ambient temperature and 800 × g. A 1.5-mL volume of the supernatant was transferred to a fresh test tube, and the extraction process was repeated. The pooled supernatants were evaporated under vacuum using a centrifugal evaporator, and the dried residues were resuspended in 100 µL of methanol-water (85:15, vol/vol). Subsequently, 10 µL of this suspension was injected into the HPLC system (Pharmacia LKB-2241). The measurement method employed was reverse-phase chromatography with a UV detector, utilizing a Super Pacpep-S column and a mobile phase of methanol-water (85:15, vol/vol) delivered at a flow rate of 1.5 mL/min. The fluorescence detector operated at an excitation wavelength of 292 nm and an emission wavelength of 325 nm. The concentration of 25-hydroxy vitamin D3 in blood serum was determined using an ELISA kit (Pishtaz Teb Zaman Diagnostics Co., Tehran, Iran) based on competitive immunoassay techniques, following the manufacturer’s instructions.

Calcium was measured by a spectrophotometric method using a Calcium CPC Liquicolor kit (Pars Azmoon Co., Tehran, Iran). Phosphorus was determined using a commercial kit (Pars Azmoon Co.), employing the molybdenum blue phosphorus method and measuring ultraviolet (UV) absorption at 340 nm. Copper was measured using the spectrophotometric method and Zn levels in serum were evaluated with the 5-Br-PAPS reagent by measuring absorption at 560 nm. Selenium was determined using Electrothermal Atomic Absorption Spectroscopy (ETAAS), and iodine was measured through indirect Atomic Absorption Spectroscopy (AAS).

### Histopathological study

A total of 62 aborted fetuses were collected from various cities within the province between November 2023 and February 2024. The aborted fetuses were categorized into four gestational age groups: 5 fetuses (8.06%) were 60–90 days old, 14 (22.58%) were 90–120 days, 36 (58.06%) were 120–150 days, and 7 (11.29%) were 150–155 days. Histopathological analysis was performed on heart and brain tissue samples from these fetuses, specifically those whose mothers had confirmed severe deficiencies in copper and selenium, as determined by serum analysis. The objective was to identify and characterize the pathological changes associated with these deficiencies [18].

To investigate the histopathological lesions, tissue samples were collected from the heart and brain of the aborted fetuses. Tissue samples were fixed in a 10% neutral buffered formalin solution for at least 48 hours. The processing of the tissues was performed using a DS2080/H tissue processor (Didsabz, Iran). Tissue samples underwent dehydration using ethanol, clarification with xylene, embedding in paraffin, cutting into 5 µm thick sections, and staining routinely with hematoxylin and eosin.

### Statistical analysis

Data are presented as means ± standard error of the mean (SEM). Additionally, the results tables include the minimum and maximum values measured for each vitamin and mineral to provide a comprehensive overview of the data distribution.

## Results and discussion

The assessment of nutritional deficiencies in the surveyed cities reveals significant shortcomings in most essential nutrients. The flocks were visited only once during sampling, and no severe clinical signs (such as fever, anorexia, or ataxia) were observed or reported by farmers. However, wool loss, wool-eating syndrome, and occasional pica were noted, all suspected to be linked to deficiencies in essential nutrients including copper, zinc, and phosphorus, which were confirmed by serum sample analysis.

### Concentrations of serum vitamins A, D, and E

The results indicate that while the majority of serum vitamin A concentrations in sheep and goat flocks are within the normal range, a notable deficiency exists in some portion of the samples. Specifically, 15.54% of the samples were found to be deficient in vitamin A, with Bostan Abad showing the highest deficiency rate at 35.13% (Table 1). Vitamin A, particularly in the form of retinol, is crucial for various physiological functions, including vision, immune response, and reproduction [12]. Deficiencies in this vitamin can lead to numerous health issues, such as increased susceptibility to infections, poor growth, infertility, abortion, retained placenta, blind fetuses, and irregularities in the reproductive cycle [19–21]. In this study, vitamin A deficiency observed in some flocks may be contributing to increased abortion rates. This finding aligns with results reported in Sakiz ewes in western Turkey [16] and underscores the importance of vitamin A supplementation in enhancing reproductive efficiency and profitability in both ewes [22] and goats [23]. The high deficiency rate in Bostan Abad in the current study is particularly concerning and suggests that there may be environmental or dietary factors in this region leading to inadequate vitamin A levels in the feed or forage consumed by the animals. Addressing these deficiencies requires targeted strategies, such as improving the nutritional content of feed, providing vitamin A supplements, and monitoring the health and reproductive outcomes of the animals.

**Table 1:** Serum concentrations of vitamins A, D, and E in sheep and goat flocks associated with abortion incidents in East Azerbaijan Province, Northwest Iran.

City	Vitamin A (µg/dL) Ref: > 22.5	Vitamin E (µg/dL) Ref: > 1	Vitamin D (ng/dL) Ref: > 30
Bostanabad			
Mean ± SEM	28.88 ± 1.84	1.43 ± 0.09	**14.05 ± 1.45**
Min–Max	14.23–54.14	1.00–3.46	4.90–32.46
Charuymaq			
Mean ± SEM	36.82 ± 1.19	2.42 ± 0.11	**22.64 ± 1.32**
Min–Max	10.00–77.78	0.99–6.73	1.76–67.1
Hashtrud			
Mean ± SEM	28.65 ± 1.18	1.70 ± 0.11	**16.68 ± 2.20**
Min–Max	17.74–42.14	1.00–2.85	3.66–50.10
Heris			
Mean ± SEM	38.10 ± 2.34	1.97 ± 0.13	**16.92 ± 1.64**
Min-Max	20.98–60.78	1.08–2.93	7.30–34.87
Jolfa			
Mean ± SEM	35.15 ± 2.21	4.28 ± 0.55	**11.10 ± 0.92**
Min–Max	12.23–57.38	1.02–12.03	4.40-21.00
Khoda Afarin			
Mean ± SEM	45.69 ± 3.03	5.43 ± 0.61	**14.39 ± 2.07**
Min–Max	23.69–71.08	2.20–9.37	5.00–33.50
Marand			
Mean ± SEM	38.83 ± 2.59	5.39 ± 0.48	**27.79 ± 3.35**
Min–Max	17.31–47.67	2.94–8.18	6.50–83.50
Mianeh			
Mean ± SEM	33.68 ± 1.29	2.09 ± 0.10	**25.22 ± 1.79**
Min–Max	5.51–58.21	0.80–4.00	5.80–100.10
Tabriz			
Mean ± SEM	33.95 ± 2.61	3.65 ± 0.32	36.13 ± 6.68
Min–Max	17.31- 47.67	2.49–6.29	6.50–83.50
Overall			
Mean ± SEM	35.02 ± 0.64	2.63 ± 0.09	**20.71** **± 0.75**
Min–Max	5.51–77.78	0.80–12.03	1.76–100.10

Values below the reference range are bolded.

The results clearly indicate that vitamin D deficiency is a widespread issue among sheep and goat flocks in the surveyed regions (Table 1). The fact that all samples from Jolfa showed deficiencies (Table 1) is particularly alarming. Environmental factors, such as limited sunlight exposure essential for synthesizing vitamin D in the skin, can significantly contribute to vitamin D deficiencies. Additionally, dietary insufficiencies resulting from inadequate vitamin D content in feed or forage may also be a factor [12]. Given the climate of the studied areas, particularly Jolfa, where sunlight exposure is generally sufficient, the first potential cause seems unlikely. However, dietary insufficiency is more likely. Animal feeds, cereals, roots, and oilseeds, together with many of their by-products, are poor in vitamin D [24]. Esmaeili et al. (2022) found that vitamin D supplements may reduce abortion rates in sheep, indicating its significance in reproductive health, likely due to improved nutritional status and immune function [4]. Recent surveys highlight the importance of vitamin D3 in the reproductive processes of humans and animals [25]. The presence of vitamin D3 receptors in female reproductive organs like the ovaries, fallopian tubes, uterus, placenta, endoderm, and trophectoderm underscores its importance during implantation and early pregnancy [26–28]. Additionally, a human study suggests that adequate vitamin D levels might reduce the risk of spontaneous abortion, although further research is needed to confirm this protective effect [29].

In the current study, in most cities, serum samples showed adequate levels of vitamin E, averaging only 12.5% below the normal range (Table 1). This suggests that while there is a slight deficiency, it is relatively marginal. This situation largely arises from farmers’ preference for using injectable forms of vitamin E during the final weeks of pregnancy in ewes. As a result, the herds studied in this project demonstrate a fairly acceptable vitamin E status compared to their levels of other vitamins and minerals. The supplementation practices adopted by farmers significantly contribute to this positive outcome. In support of this, a previous study has shown that vitamin E supplementation significantly increased serum α-tocopherol levels, which were associated with a reduced stillbirth rate in ewes with three or more lambs [30].

### Concentrations of serum macroelements

In the present study, the most striking finding was the 100% deficiency rate of calcium in Bostan Abad, indicating that every individual sampled in this city lacked adequate calcium intake, which was particularly concerning. Other cities, such as Charuymaq (88.63%) and Khoda Afarin (94.73%), also exhibited high deficiency rates, suggesting that calcium intake was a widespread issue across the region (Table 2). Phosphorus deficiency was observed at an overall rate of 33.24% below the reference range, with Khoda Afarin exhibiting the highest deficiency rate at 57.89%. However, in cities such as Tabriz, Marand, and Hashtrud, more than 80% of the samples were not phosphorus deficient (Table 2). The data clearly indicate an insufficient supply of calcium to meet the demands of pregnancy and lactation. In pregnant ewes, calcium needs rise significantly late in pregnancy due to the expanding fetus, leading to hypocalcemia from insufficient dietary calcium typically occurring 2–6 weeks before lambing. Calcium deficiencies can result in poor bone development in growing lambs [31,32]. In most of the studied areas, from spring to mid-autumn, sheep and goats grazed on poor pastures, particularly fast-growing spring grass. This situation resulted from several years of drought and significant grazing pressure from livestock, leading to the rapid depletion of grasslands. During the winter months, the animals were kept indoors and fed a diet primarily composed of harvested forages from pastures, crop residues, and cereal grains (mainly wheat or barley) without adequate calcium or phosphorus supplementation. The relatively better status of serum phosphorus compared to calcium might be due to the consumption of cereal grains, which are good sources of phosphorus. Calcium and vitamin D signaling are crucial for establishing pregnancy and regulating fetal and placental growth [28,33]. Insufficient phosphorus intake can compromise immune systems, increasing the risk of abortions and reproductive issues [12,34,35], as previously reported in ewes and goats in South Khorasan province, Iran [36], and in Sakız sheep in Western Turkey [16].

**Table 2:** Serum concentrations of macroelements and trace elements in sheep and goat flocks associated with abortion incidents in East Azerbaijan Province, Northwest Iran.

City	Calcium (mg/dL) Ref: 11.5–12.8	Phosphorous (mg/dL) Ref: 5–7.3	Copper (µg/dL) Ref: 60–100	Zinc (µg/dL) Ref: 100–120	Iodine (µg/dL) Ref: > 40	Selenium (µg/dL) Ref: > 110
Bostan Abad						
Mean ± SEM	**9.49 ± 0.15**	5.89 ± 0.24	64.02 ± 1.92	**64.82** ± 3.31	40.73 ± 0.80	**55.71 ± 15.19**
Min–Max	6.80–11.10	2.80–10.00	45.00–87.00	31.00–111.00	29.99–51.02	13.39–128.34
Charuymaq						
Mean ± SEM	**9.93 ± 0.10**	5.72 ± 0.11	63.69 ± 1.92	**74.80** ± 3.00	**35.13 ± 1.98**	**36.73 ± 2.54**
Min–Max	7.20–12.80	2.90–11.10	6.70–229.00	34.00–304.00	19.09–279.78	19.09–139.83
Hashtrud						
Mean ± SEM	**10.62 ± 0.29**	5.93 ± 0.16	**54.76** ± 2.31	**67.76 ± 4.87**	47.94 ± 0.78	**32.70 ± 2.32**
Min–Max	8.40–14.40	4.10–7.50	42.00–94.00	38.00–149.00	39.89–55.21	17.18–56.82
Heris						
Mean ± SEM	**9.73 ± 0.25**	7.05 ± 1.81	66.23 ± 2.29	**72.23 ± 3.62**	**38.90 ± 0.81**	**40.24 ± 1.20**
Min–Max	8.40–12.90	3.40–43.00	55.00–93.00	48.00–108.00	29.87–45.21	30.91–50.62
Jolfa						
Mean ± SEM	**9.79 ± 0.22**	5.84 ± 0.25	61.96 ± 2.10	**77.90** **± 4.75**	**38.80 ± 0.72**	**41.91 ± 1.57**
Min–Max	7.90–12.60	3.10–9.20	45.00–92.00	31.00–148.00	30.56–48.11	19.70–63.25
Khoda Afarin						
Mean ± SEM	**9.73 ± 0.27**	5.07 ± 0.26	61.13 ± 2.99	**73.93** **± 4.11**	**30.53 ± 0.93**	**44.15 ± 5.13**
Min–Max	7.90–12.20	2.50–6.70	42.00–80.00	50.00–104.00	21.09–36.12	15.86–69.12
Marand						
Mean ± SEM	**9.58 ± 0.36**	6.31 ± 0.25	67.82 ± 6.29	102.87 ± 10.62	**22.48 ± 0.52**	**48.62 ± 2.25**
Min–Max	7.70–12.30	4.00–7.70	7.40–105.00	68.00–225.00	19.81–26.16	34.59–60.66
Mianeh						
Mean ± SEM	**10.23 ± 0.12**	5.49 ± 0.12	61.65 ± 1.67	**64.90** **± 2.56**	46.44 ± 0.46	**48.96 ± 3.34**
Min–Max	8.20–12.70	3.80–8.20	38.00–98.00	24.00–154.00	39.78–56.11	1.64–122.86
Tabriz						
Mean ± SEM	**9.41 ± 0.34**	6.66 ± 0.32	72.81 ± 9.09	**99.12** **± 6.75**	**23.18 ± 0.67**	**84.70 ± 3.51**
Min–Max	7.70–12.30	2.50–43.00	35.00–194.00	58.00–142.00	19.82–29.98	69.97–97.05
Overall						
Mean ± SEM	**9.97 ± 0.06**	5.83 ± 0.12	63.17 ± 0.98	**73.86** **± 1.54**	**38.09 ± 0.82**	**42.89 ± 1.42**
Min–Max	6.80–14.40	2.50–43.00	6.70–229.00	24.00–304.00	19.09–279.78	1.64–139.83

Values below the reference range are bolded.

### Concentrations of serum trace-elements

**Fig 1:**
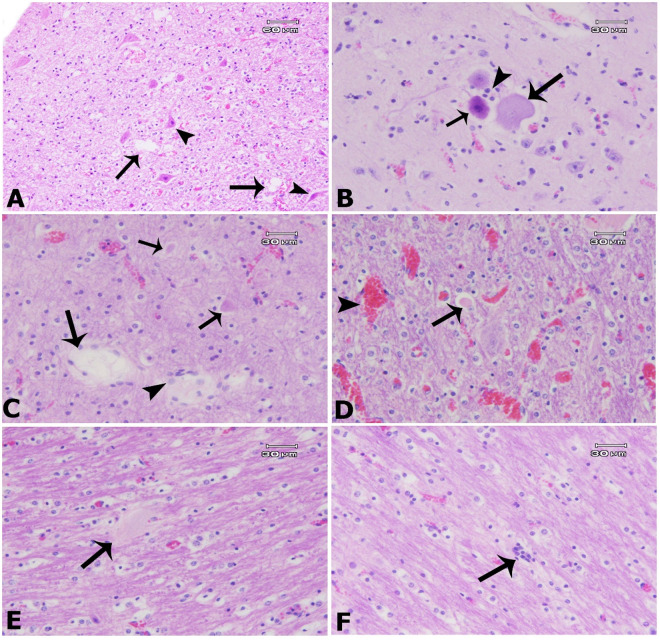
The brain (cerebellum) of an aborted fetus from a mother with confirmed copper deficiency shows various pathological features. A: Encephalomalacia (vacuolated spaces, long arrows) and neuronal necrosis (shrunken, angular neuronal cell bodies with dense, contracted nuclei, short arrows). B: Central chromatolysis (neuron with an acentric nucleus, pale central cytoplasm, and dispersed Nissl substance, long arrow), neuronal necrosis (short arrow), and satellitosis (oligodendrocyte proliferation, arrowhead). C: Encephalomalacia (long arrow) associated with Wallerian degeneration (swollen axons, short arrows) and foamy macrophage infiltration (gitter cells with distended, lipid-rich cytoplasm). D: Wallerian degeneration (swollen axons, arrow) and vascular hyperemia. E: Wallerian degeneration (swollen axons, longitudinal section, arrow). F: Satellitosis (oligodendrocyte proliferation in small groups within the interfascicular space, arrow; H&E stain).

The analysis of nutritional deficiencies in sheep and goat flocks has revealed a significant lack of copper (Table 2). In most cities, around 40–50% of the samples were deficient in copper; however, in Hashtrud, 76% of the samples were below the normal range. This deficiency is particularly prevalent in regions experiencing high rates of abortion among these animals. Insufficient copper intake during this critical period can result in higher rates of abortion, stillbirths, and weak offspring with compromised health [22]. It seems that the primary cause of copper deficiency in the current study is the poor quality of pastures and forage, which are expected to be low in essential minerals due to prolonged drought conditions and overgrazing. Additionally, the use of trace mineral salt blocks in the pastures studied is uncommon, and during the winter period, when the animals are kept indoors, the use of mineral or vitamin supplements is not part of the farmers’ feeding programs. Copper deficiency in ewes and goats experiencing abortion has previously been reported in South Khorasan province, Iran [36]. Symptoms of copper deficiency include anemia, reduced appetite and growth, bone disorders, cardiovascular problems, poor wool quality, infertility leading to small or stillborn offspring, and neonatal ataxia, commonly referred to as “swayback” or “lamkruis” [13,37,38]. This neurological disorder results in an imbalance in lambs and can lead to high mortality rates [39,40]. Wool loss and wool-eating syndrome observed in many sheep flocks in this study are believed to be associated with deficiencies in microelements, particularly copper and zinc [41]. In the brains of 21 out of 62 aborted fetuses from mothers with confirmed copper deficiencies in the present study, we observed central chromatolysis (neurons with acentric nuclei, pale central cytoplasm, and peripherally dispersed Nissl substance), neuronal necrosis (shrunken, angular neuronal cell bodies with dense nuclei), encephalomalacia, Wallerian degeneration (indicating axonal injuries and demyelination), and satellitosis (hypertrophy and proliferation of oligodendrocytes due to neuronal and axonal injuries), particularly in the cerebellar white matter. Additionally, foamy macrophages (gitter cells) accumulated in necrotic areas, characterized by distended, lipid-rich cytoplasm (Fig 1). In healthy conditions, oligodendrocytes in white matter are typically arranged in linear patterns and myelinate axons. In this study, oligodendrocytes exhibited hypertrophic changes and clustered arrangements (Fig 1). These histopathological changes are associated with copper deficiency [18]. Interactions within the digestive system of ruminants, particularly concerning copper and other dietary elements such as molybdenum, sulfur, zinc, and iron, significantly affect the availability of dietary copper [13]. Maintaining balanced copper levels in livestock diets is essential to prevent production and reproductive issues. Both copper deficiency and toxicity raise serious concerns for sheep’s reproductive health [13,37]. Research has shown that deficiencies in certain vitamins and minerals, including zinc and vitamin A, can exacerbate the effects of copper deficiency on reproductive health [42].

The study reveals significant differences in zinc levels across various cities, demonstrating a widespread deficiency in sheep and goat flocks (Table 2), which might be associated with higher instances of abortion. Apart from Marand and Tabriz, where 53.33% and 57.14% of the samples were zinc deficient, respectively, the majority of the surveyed cities exhibited deficiencies in over 85% of the samples. Similar potential causes discussed for copper deficiency appear to apply to zinc as well. Research indicates that approximately 50% of the world’s soils could be deficient in zinc. This deficiency can lead to plants grown in those soils having low zinc levels, which are subsequently consumed by livestock [43]. Serum zinc deficiency has previously been reported in ewes [16] and goats [44] that experienced abortion, compared to healthy controls in other countries. However, in contrast to our findings, Omıdı (2015) did not find significant differences in serum zinc levels between healthy and aborted ewes in South Khorasan Province, Iran [36]. This discrepancy might be due to higher soil zinc levels in that region compared to East Azerbaijan Province, or it could be a result of the flocks being supplemented with zinc. Symptoms of zinc deficiency in animals may include decreased appetite, growth, and reproductive issues, bone health problems, hair loss, skin lesions, deformed hooves, and swollen joints [13]. In sheep, zinc deficiency can cause wool to become loose, brittle, and shed [13,45], also contributing to wool-eating syndrome [41]; such issues were observed in many sheep flocks during this study. Zinc is essential for antioxidant defense, growth, reproduction, and immune response in both animals and humans [46]. Studies indicate that zinc supplements can enhance reproductive outcomes, like lamb production, by improving sperm quality and protecting against free radicals [47,48]. Maternal zinc deficiency can lead to infertility, fetal death, slow growth, and congenital defects, with post-birth effects including behavioral disorders and immune deficiencies, posing risks during fetal development [49–51].

Goiter and thyroid issues in sheep and goats due to iodine deficiency have also been reported [52]. In regions with endemic human goiter, widespread thyroid lesions in ewes and fetuses have been observed [53]. A 2009 study showed significant differences in iodine status in sheep across various regions and seasons in Markazi [54]. Low-iodine diets in ewes (less than 0.5 micrograms/kg body weight per day) lead to higher abortion rates [55]. Research by Abozed et al. demonstrated that iodine supplementation improves physiological responses, metabolic rate, and blood components in pregnant Saedi ewes, leading to better performance and viability of lambs [56]. In the current study, the differences in iodine deficiency levels among regions emphasize the need for targeted interventions in Marand and Khoda Afarin. Strategies could include dietary iodine supplementation, improved pasture management, and regular monitoring of iodine levels in livestock.

Iodine deficiency was particularly high in Marand and Khoda Afarin (100%), whereas only 4–6% of the blood samples in Hashtrud and Mianeh fell below the reference range. Low iodine levels in feed, affected by soil content [57] and distance from the sea [58], may impact thyroid as well as reproductive function [59], as indicated by increased abortion and stillbirth rates in iodine-deficient sheep [60–63]. Reports from other regions of the country support similar findings regarding thyroid dysfunction in sheep [52–54,64,65]. Moreover, low iodine intake (<0.5 μg/kg/day) increases abortion rates [55], while supplementation enhances physiological responses and lamb viability in pregnant Saedi ewes [56].

**Fig 2:**
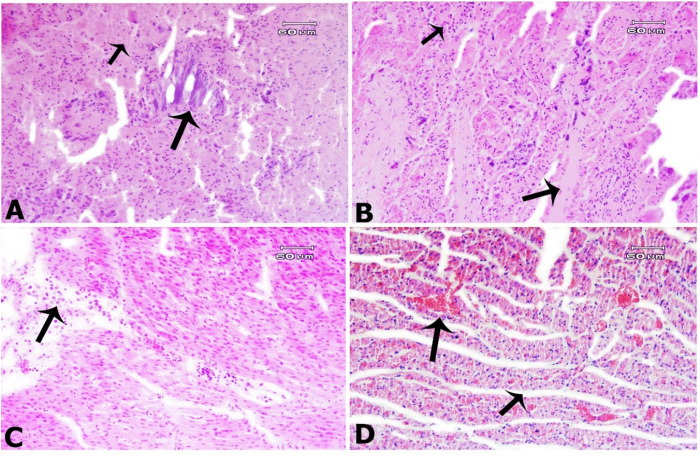
The myocardium of an aborted fetus from a mother with confirmed selenium deficiency exhibits several pathological features. A: Calcium deposits (long arrow) and myocardial necrosis (short arrow) are observed. B: Myocardial cell hyalinization (long arrow) is accompanied by satellite cell proliferation (short arrow). C: Macrophage infiltration (arrow) is present in the damaged myocardium. D: Hemorrhage (long arrow) and edema (short arrow) occur between myocardial bundles. H&E.

Serum selenium was deficient in over 87% of the samples (Table 2), a trend observed across many regions in Iran [66]. Selenium deficiency is associated with conditions like white muscle disease in pregnant sheep [67,68] and cardiac complications in aborted fetuses [69]. In our study, myocardial histopathological changes, including significant fragmentation and hyalinization of myocardial cells, along with hemorrhage, edema between myocardial bundles, focal calcification, macrophage infiltration, satellite cell proliferation, and myocardial necrosis in the heart sections were observed in two aborted fetuses (Fig 2), which are consistent with selenium deficiency [18]. Selenium supplementation during late pregnancy improves blood levels, antioxidant status, and reproductive health [70,71]. Despite the use of injectable vitamin E and selenium during late pregnancy, most herds remained deficient, suggesting issues with soil composition, mineral bioavailability, and the timing or dosage of supplementation.

## Conclusion

This study highlights critical deficiencies in essential vitamins and minerals for sheep and goat health in the examined regions. While serum vitamin A levels are mostly normal, significant deficiencies exist, especially in Bostan Abad. Vitamin D deficiency is prevalent, notably in Jolfa, indicating potential dietary shortcomings despite adequate sunlight. A slight vitamin E deficiency was noted, somewhat addressed by farmers’ late pregnancy supplementation. However, calcium and phosphorous levels, particularly calcium, raise more serious concerns, alongside widespread copper and zinc deficiencies. Brain sections from the aborted fetuses of mothers with confirmed copper deficiency showed severe degeneration. Iodine deficiency is particularly problematic in Marand and Khoda Afarin, impacting thyroid and reproductive health. Selenium deficiency is notably high, affecting over 87% of samples, stressing the need for proper supplementation during pregnancy. Pathological findings in the aborted fetuses of mothers with confirmed selenium deficiency revealed severe myocardial cell damage. These results highlight the necessity for specific nutritional strategies, regular monitoring, and effective supplementation to enhance livestock health and productivity in the studied regions. Improvements can be achieved through dietary changes and balanced supplementation, considering local factors like soil and forage quality.

## Supporting information

S1 Dataset Raw serum nutrient measurements for each sampled animal. Spreadsheet includes columns for City code, Ca, P, Cu, Zn, I, Se, and vitamins A, D, and E.
(XLSX)

S1 Table Characteristics of the 43 sheep and goat flocks sampled. (PDF)
