# Supplementary material for: Nutritional deficiencies and abortions in sheep and goats: An in-depth study from East Azerbaijan Province, Northwest Iran
Source: PLoS One. 2025 Aug 5;20(8):e0327768. doi: 10.1371/journal.pone.0327768 (PMC12324140; doi:10.1371/journal.pone.0327768)
Supplement: S1 Table — (PDF) [file pone.0327768.s002.pdf]

S1 Table. Characteristics of the 43 sheep and goat flocks sampled.

City	Flock number	Herd size	Abortion rate%
Tabriz	1	500	2.4
	2	100	5
	3	125	8.8
	4	50	14
	5	85	3.5
	6	300	1.5
Marand	1	110	21.81
Charuymaq	1	290	7.5
	2	355	7.04
	3	200	5
	4	150	4.66
	5	260	3.1
	6	350	4.28
	7	105	5.71
	8	243	12.34
	9	320	8.12
	10	60	5
	11	40	8
	12	68	13.23
	13	100	6
	14	510	0.4
	15	80	2.5
	16	200	0.5
Khoda Afarin	1	130	24.61
	2	370	17.83
	3	110	19.1
Jolfa	1	700	5.71
	2	730	2.75
Heris	1	85	35.3
Bostan Abad	1	396	9.6
	2	220	4.54
	3	50	6
	4	169	3.55
	5	52	38.46
	6	355	5.66
	7	35	2.85
	8	130	2.3
Mianeh	1	236	17.8
	2	220	25
	3	75	42.6
	4	300	26.6
	5	150	10
Hashtroud	1	132	62.12
